# Hepatic failure associated with immune checkpoint inhibitors: An analysis of the Food and Drug Administration Adverse Event Reporting System database

**DOI:** 10.1002/cam4.5655

**Published:** 2023-02-03

**Authors:** Ye Xu, Cilin Yan, Ying Zhao, Dandan Li, Mingxing Guo, Xiangli Cui

**Affiliations:** ^1^ Department of Pharmacy, Beijing Friendship Hospital Capital Medical University Beijing China; ^2^ School of Pharmacy Capital Medical University Beijing China; ^3^ School of Automation Science and Electrical Engineering Beihang University Beijing China

**Keywords:** adverse event reporting system, cytotoxic T lymphocyte‐associated protein 4, hepatic failure, immune checkpoint inhibitors, programmed cell death 1 ligand 1, programmed cell death protein 1

## Abstract

**Background:**

Hepatic failure induced by immune checkpoint inhibitors (ICIs) has been reported in only a few case series and case reports.

**Objective:**

We aimed to explore the association between ICIs and hepatic failure and characterize the clinical features of ICI‐associated hepatic failure in the pharmacovigilance database.

**Methods:**

Data from the first quarter (Q1) of 2015 to the fourth quarter (Q4) of 2021 in the US Food and Drug Administration Adverse Event Reporting System (FAERS) database were retrieved for disproportionality and Bayesian analysis. Reporting odds ratios (ROR) and information component (IC) were used to evaluate correlations between ICIs and hepatic failure.

**Results:**

Hepatic failure occurred in 0.19% (18,454/9,647,655) of all cases in the FAERS database, of which 654 cases were associated with ICIs. The overall median time from ICIs initiation to hepatic failure onset was 38 days, 72.3% of the adverse events occurred within the first 3 months, and 68.65% of the cases died after developing hepatic failure. In general, a strong signal was shown between ICIs and hepatic failure (ROR_025_ = 2.70, IC_025_ = 1.39). For the three categories of ICIs, programmed cell death 1 ligand 1 inhibitors (ROR_025_ = 3.09, IC_025_ = 1.57) had a higher risk signal than programmed cell death protein 1 inhibitors and cytotoxic T lymphocyte‐associated protein 4 inhibitors. For monotherapy, atezolizumab showed the strongest risk signal (ROR_025_ = 4.07, IC_025_ = 1.90). The combination of nivolumab and ipilimumab showed stronger signals of hepatic failure compared with nivolumab or ipilimumab alone (nivolumab + ipilimumab vs. ipilimumab: ROR_025_ = 1.40, IC_025_ = 0.16; nivolumab + ipilimumab vs. nivolumab: ROR_025_ = 1.24, IC_025_ = 0.34). Considering the concomitant agents used with ICIs, the majority of these regimens showed stronger signals than ICI monotherapy, such as acetaminophen (ICIs + acetaminophen vs. ICIs: ROR_025_ = 1.06, IC_025_ = 0.32).

**Conclusions:**

ICIs had possible strong signals associated with hepatic failure, and most cases of hepatic failure occurred within the first 3 months and had poor outcomes, which should attract clinical attention.

## Graphical abstract

This study aimed to explore the association between ICIs and hepatic failure. Data from the FAERS database were analyzed for Reporting odds ratios (ROR) and information component (IC).

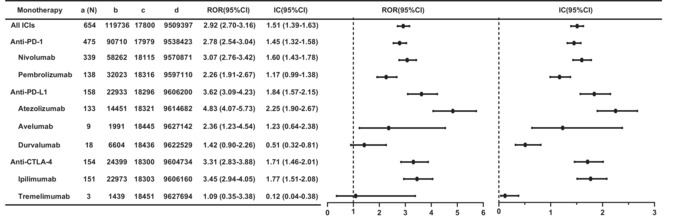

## 1 INTRODUCTION

Immune checkpoint inhibitors (ICIs) have been approved to treat melanoma, urothelial carcinoma, non‐small cell lung cancer, hepatocellular carcinoma, and so on, and have largely improved the survival of patients with advanced malignancy.^1^ Currently approved ICIs are mainly classified into three categories: Programmed cell death protein 1 (PD‐1) inhibitors, programmed cell death 1 ligand 1 (PD‐L1) inhibitors, and cytotoxic T lymphocyte‐associated protein 4 (CTLA‐4) inhibitors. With the wide use of ICIs, reports of adverse effects are gradually increasing and are commonly found in the skin, respiratory system, heart, and liver.^2^

Although most ICI‐associated liver damage usually presents as mild elevations of liver enzymes, hepatic failure induced by ICIs is rare but potentially life‐threatening.^3^, ^4^ Recently, some case reports have raised awareness of hepatic failure induced by ICIs.^5^, ^6^, ^7^ A systematic review concluded that the incidence of grade 3 and 4 liver damage induced by ICIs was 0.6%–11% and the incidence of hepatic failure was 0.1%–0.2%.^3^ A pharmacovigilance study based on the WHO VigiBase database reported 613 ICI‐associated fatal adverse events from 2009 to January 2018, of which 124 (20.2%) cases were associated with liver damage.^8^

ICIs activate T cells to exert anti‐tumor effects by competitively inhibiting PD‐1, PD‐L1, and CTLA‐4 targets; however, this can also lead to immune‐related adverse events,^9^ of which hepatic failure is a serious event.^10^ The mechanisms of hepatic failure induced by ICIs are unclear but may include the following three factors: (1) over‐activated T cells attacking healthy liver tissue; (2) increased inflammatory cytokines, such as interleukin (IL)‐1a, IL‐2, and interferon (IFN)‐α2, disrupting the homeostatic immunity; and (3) increased autoantibodies contributing to a phenomenon similar to autoimmune hepatitis (AIH), with the difference that autoantibodies are generally low or negative.^11^

There is a lack of specific medications and treatments for hepatic failure. Liver transplantation is the most effective treatment, but donor livers are scarce, and the procedure is expensive. Therefore, early prediction of the risk of hepatic failure and timely intervention are very important. However, the detailed characteristics of ICI‐associated hepatic failure have not been thoroughly investigated, and the differences in risk between different ICI regimens are still controversial. The impact of combinations with other drugs on ICI‐associated hepatic failure is also still unknown. In addition, data from clinical trials might not comprehensively represent the real world, and only a few cases reported in the literature were inadequate to characterize hepatic failure. Therefore, we have contributed in a small way to solving the above problems based on the US Food and Drug Administration Adverse Event Reporting System (FAERS) database,^12^ by exploring the association between ICIs and hepatic failure, as well as analyzing the factors impacting hepatic failure.

## 2 MATERIALS AND METHODS

### 2.1 Data sources

Data were retrieved based on the FAERS database from the first quarter (Q1) of 2015 to the fourth quarter (Q4) of 2021. The FAERS database is a spontaneous reporting system, and adverse events are reported by physicians, consumers, health professionals, and pharmacists from different regions. FAERS data files contain demographic and administrative information, drug information, preferred terms (PTs) coded for the adverse event, patient outcomes, report sources, therapy start dates and end dates for reported drugs, and indications for use. The FAERS database is free and publicly available at the website (shown in the data source): https://open.fda.gov/data/faers/.

### 2.2 Study design

In the FAERS database, adverse event reports were coded by PTs from Standardized MedDRA Queries (SMQ) of Medical Dictionary for Regulatory Activities (MedDRA, Version 23.0). We considered the following mutually exclusive PTs as related to hepatic failure: “hepatic failure [10019663],” “acute hepatic failure [10000804]”, “subacute hepatic failure [10056956],” “acute on chronic liver failure [10077305],” and “chronic hepatic failure [10057573].”

Both generic names and brand names were used to identify 8 FDA approved ICIs (Table S1). ICIs can be administered as monotherapy or combination therapy. Monotherapy was identified when a specific ICI was reported as the “primary suspect” or “secondary suspect.” Combination therapy was the administration of two or more ICIs, identified when a specific ICI was reported as the “primary suspect” and the other ICIs were reported as the “secondary suspect.”

We removed duplicated records by selecting the latest FDA_DT when the CASEID and FDA_DT were the same. Among the retrieved reports of ICI‐associated hepatic failure, we extracted information including age, gender, reporting country, reporting region, reporting year, reporter type, indication, drug name, and outcome. The time to onset (TTO) of hepatic failure for different ICIs, defined as the interval time between the adverse event onset date and the first dose date, were also evaluated. Additionally, we calculated the fatality proportion from the total number of ICI‐associated hepatic failures.

### 2.3 Outcomes

#### 2.3.1 Primary outcomes

In order to estimate the association between ICIs and hepatic failure, we designed four comparison groups including: (1) comparing different categories of ICIs with all other drugs in the full database; (2) comparing ICI monotherapy with all other drugs; (3) comparing ICI combination therapy with ICI monotherapy; and (4) comparing combinations of ICIs and the most frequently used concomitant agents with ICI monotherapy.

#### 2.3.2 Secondary outcomes

Subgroup analyses were also conducted, including female versus male; and younger group (age < 65 years) versus older group (age ≥ 65 years). We only calculated the ROR when comparing disproportionality signal differences in different ICI subgroups.

### 2.4 Statistical analysis

We used descriptive analysis to summarize the clinical features of ICI‐associated hepatic failure. Reporting odds ratio (ROR) and information component (IC), two specific indices of disproportionality analysis, were calculated to identify potential signals. Strong associations were defined when the lower limit of the 95% two‐sided confidence interval of the ROR (ROR_025_) was greater than 1 with at least 3 cases, or the lower limit of the 95% two‐sided confidence interval of the IC (IC_025_) was greater than 0. The equations of these two algorithms are listed in Table S2.^13^, ^14^ All data were processed using Python (Version 3.7.0).

## 3 RESULTS

### 3.1 Descriptive analysis

Hepatic failure occurred in 0.19% (18,454/9,647,655) of all cases in the FAERS database, of which 654 cases were associated with ICIs (Figure S1), and the clinical characteristics of these patients are presented in Table 1. Males (*n* = 382, 58.41%) had a larger proportion of hepatic failure than females (*n* = 218, 33.33%), and gender information was unavailable for 54 patients. The largest proportion of cases was aged 45–64 years (*n* = 215, 32.87%), with a median age of 64 (range 14–90) years. Most reports were from Europe (*n* = 221, 33.79%), Asia (*n* = 215, 32.87%), and North America (*n* = 184, 28.13%), with the number of cases increasing from 2015 to 2021, and more than one‐half of these cases were reported by physicians (*n* = 330, 50.46%). The most common indication for ICIs was lung cancer (*n* = 118, 18.04%). Only 5 cases (*n* = 5, 0.76%) had liver metastasis, with 1 case of metastatic esophageal adenocarcinoma to the liver, 1 case of metastatic lung malignancy to the liver, 1 case of metastatic kidney cancer to the liver, and the other 2 cases had unknown primary disease. Of the three categories of ICIs, anti‐PD‐1s (*n* = 475, 72.63%) were reported more than anti‐PD‐L1s (*n* = 158, 24.16%) and anti‐CTLA‐4s (*n* = 154, 23.55%). Among all ICIs, nivolumab was associated with the highest number of cases (*n* = 339, 51.83%), followed by ipilimumab (*n* = 151, 23.09%) and pembrolizumab (*n* = 138, 21.10%). The combination of nivolumab and ipilimumab was most frequently reported in ICI combination therapy (*n* = 126, 95.45%).

**TABLE 1:** Clinical characteristics of patients with ICI‐associated hepatic failure in the FAERS database.

Characteristics	*N*	%
All ICIs	654	
Gender		
Male	382	58.41%
Female	218	33.33%
Unknown or missing	54	8.26%
Age at onset (years)		
Median age at onset (range)	64 (14–90)	
<18	2	0.31%
18–44	57	8.72%
45–64	215	32.87%
65–74	164	25.08%
≥75	87	13.30%
Unknown or missing	129	19.72%
Reporting region		
Europe	221	33.79%
Asia	215	32.87%
North America	184	28.13%
Oceania	19	2.91%
south and Central America	12	1.83%
Africa	2	0.31%
Unknown or missing	1	0.15%
Reporting year		
2015	20	3.06%
2016	36	5.50%
2017	64	9.79%
2018	94	14.37%
2019	101	15.44%
2020	139	21.25%
2021	199	30.43%
Unknown or missing	1	0.15%
Reporter type		
Physician	330	50.46%
Consumer	119	18.20%
Health professional	91	13.91%
Other health professionals	77	11.77%
Pharmacist	33	5.05%
Unknown or missing	4	0.61%
Time to onset (days)		
Median time to onset (range)	38 (1–914)	
≤28	113	38.18%
29–56	64	21.62%
57–84	37	12.50%
>84	82	27.70%
Unknown or missing	358	‐
Indications		
Lung cancer	118	18.04%
Malignant melanoma	107	16.36%
Hepatocellular carcinoma	94	14.37%
Renal cell carcinoma	55	8.41%
Breast cancer	18	2.75%
Unknown or missing		
Outcome		
Death	449	68.65%
Hospitalization	89	13.61%
Other Serious	81	12.39%
Life threatening	26	3.98%
Disability	8	1.22%
Unknown or missing	1	0.15%
Monotherapy		
Anti‐PD‐1s	475	72.63%
Nivolumab	339	51.83%
Pembrolizumab	138	21.10%
Cemiplimab	1	0.15%
Anti‐PD‐L1s	158	24.16%
Atezolizumab	133	20.34%
Avelumab	9	1.38%
Durvalumab	18	2.75%
Anti‐CTLA‐4s	154	23.55%
Ipilimumab	151	23.09%
Tremelimumab	3	0.46%
Combination therapy		
Nivolumab + Ipilimumab	126	95.45%
Durvalumab + Tremelimumab	3	2.27%
Pembrolizumab + Atezolizumab + Nivolumab + Durvalumab	2	1.52%
Pembrolizumab + Ipilimumab + Nivolumab + Atezolizumab	1	0.76%
Number of concomitant agents		
<3	384	58.72%
3–5	101	15.44%
6–10	97	14.83%
>10	72	11.01%

Abbreviations: CTLA‐4s: cytotoxic T lymphocyte‐associated protein 4, ICIs: Immune checkpoint inhibitors, PD‐1s: programmed cell death protein 1, PD‐L1s: programmed cell death 1 ligand 1.

**FIGURE 1:**
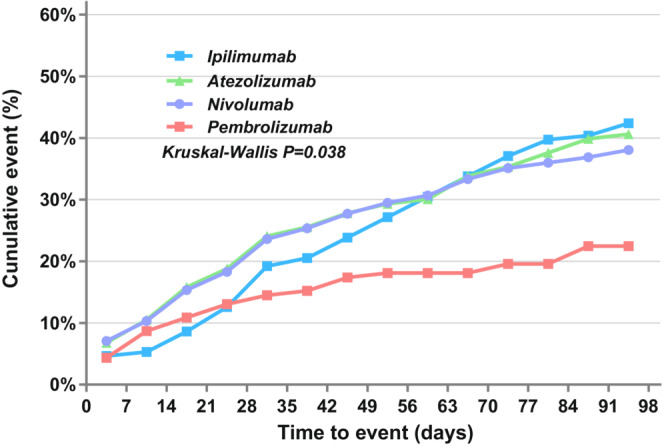
Time to onset of ICI‐associated hepatic failure.

The median TTO of hepatic failure was 38 (range 1–914) days, and 72.30% (214/296) occurred within the first 3 months. The median TTO was 29 (range 1–174) days for pembrolizumab, 35.5 (range 1–380) days for atezolizumab, 38 (range 1–863) days for nivolumab, 39 (range 2–196) days for durvalumab, 54.5 (range 1–424) days for ipilimumab, 66 (range 4–775) days for avelumab, and 172 (range 34–196) days for tremelimumab. However, we performed the Kruskal–Wallis test for two‐by‐two comparisons for groups with a case number greater than 20, and the results showed an overall significant difference in the distribution of TTO for these groups of ICI monotherapy (*p* = 0.038), but two‐by‐two comparisons showed no statistical difference (*p >* 0.05) (Figure 1).

The outcome of patients with hepatic failure was poor, with a fatality proportion of 68.65% (449/654), and the highest fatality proportion was associated with avelumab (88.89%, 8/9). Monofactor analysis was performed for ICIs with case numbers greater than 20, and Pearson's chi‐squared test showed no statistical difference (*p* = 0.282) (Table S3).

### 3.2 Disproportionality analysis and Bayesian analysis

**FIGURE 2:**
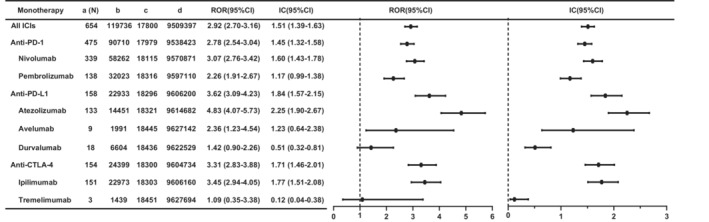
Signals of ICI‐associated hepatic failure in different ICI monotherapy. 95% CI: the 95% two‐sided confidence interval; IC: information component; ROR: reporting odds ratio.

To estimate the association between ICIs and hepatic failure, we compared different ICIs with all other drugs in the full database (Figure 2). In general, the results showed a strong association between ICIs and hepatic failure (ROR 2.92, 95% CI 2.70–3.16; IC 1.51, 95% CI 1.39–1.63). We compared different categories of ICIs with all other drugs. For the three categories of ICIs, anti‐PD‐1s (ROR 2.78, 95% CI 2.54–3.04; IC 1.45, 95%CI 1.32–1.58), anti‐PD‐L1s (ROR 3.62, 95% CI 3.09–4.23; IC 1.84, 95%CI 1.57–2.15), and anti‐CTLA‐4s (ROR 3.31, 95% CI 2.83–3.88; IC 1.71, 95% CI 1.46–2.01), all showed possible high signals. Secondly, we calculated the ROR when comparing disproportionality signal differences in these subgroups (Table S5). Anti‐PD‐L1s and anti‐CTLA‐4s had a stronger risk signal of hepatic failure compared with anti‐PD‐1s (anti‐PD‐L1s vs. anti‐PD‐1s: ROR 1.32, 95% CI 1.10–1.58; anti‐CTLA‐4s vs. anti‐PD‐1s: ROR 1.21, 95% CI 1.004–1.45), and anti‐PD‐L1s had no risk signal compared with anti‐CTLA‐4s (ROR 1.09, 95% CI 0.87–1.36).

For monotherapy (Figure 2), most ICI monotherapies showed strong signals compared with all other drugs in the full database, except for durvalumab (ROR 1.42, 95% CI 0.90–2.26; IC 0.51, 95% CI 0.32–0.81) and tremelimumab (ROR 1.09, 95% CI 0.35–3.38; IC 0.12, 95% CI 0.04–0.38). Cemiplimab was not analyzed as only 1 case was identified. Importantly, atezolizumab had the strongest risk signal (ROR 4.83, 95% CI 4.07–5.73; IC 2.25, 95% CI 1.90–2.67), followed by ipilimumab (ROR 3.45, 95% CI 2.94–4.05; IC 1.77, 95% CI 1.51–2.08) and nivolumab (ROR 3.07, 95% CI 2.76–3.42; IC 1.60, 95% CI 1.43–1.78).

**FIGURE 3:**
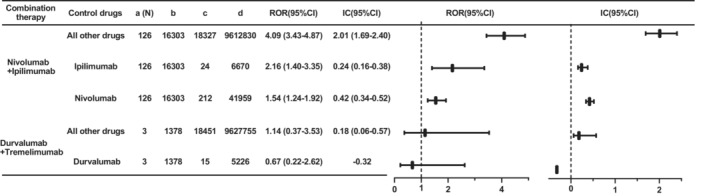
Signals of ICI‐associated hepatic failure in different ICI combination therapy. 95% CI: the 95% two‐sided confidence interval; IC: information component; ROR: reporting odds ratio.

For combination therapy, the most common therapy was the combination of nivolumab and ipilimumab, and stronger signals of this regimen were observed when compared to all other drugs (ROR 4.09, 95% CI 3.43–4.87; IC 2.01, 95% CI 1.69–2.40), ipilimumab (ROR 2.16, 95% CI 1.40–3.35; IC 0.24, 95% CI 0.16–0.38), and nivolumab (ROR 1.54, 95% CI 1.24–1.92; IC 0.42, 95% CI 0.34–0.52) (Figure 3). As for the combination of durvalumab and tremelimumab, there were only 3 cases, without a risk signal compared with durvalumab (ROR 0.67, 95% CI 0.22–2.62; IC −0.32).

To investigate which agents can increase the risk signals of ICI‐associated hepatic failure, we compared combinations of ICIs and the most frequently used concomitant agents with ICI monotherapy (Figure S2). The majority of these regimens showed strong signals, except for carboplatin (ROR 0.81, 95% CI 0.60–1.10; IC −0.27). The results showed that concomitant use of bevacizumab (ROR 3.31, 95% CI 2.61–4.21; IC 1.59, 95% CI 1.25–2.01), prednisolone (ROR 2.91, 95% CI 2.20–3.84; IC 1.45, 95% CI 1.09–1.91), and others seemed to have higher risk signals than ICIs alone.

## 4 DISCUSSION

To the best of our knowledge, this is the first study to evaluate the association between ICIs and hepatic failure based on the FAERS pharmacovigilance database. From this study, we noted several key findings. Most ICIs regimens may be associated with hepatic failure. Anti‐PD‐L1s might have a higher risk signal than anti‐PD‐1s and anti‐CTLA‐4s, and atezolizumab might show the strongest risk signals in different ICI monotherapy. Concomitant used ICIs with hepatotoxic agents, such as the combination of ICIs and acetaminophen, may be more associated with hepatic failure compared with ICIs alone. Younger patients might have a higher association with hepatic failure.

We, firstly, compared different ICIs with all other drugs in the full database. For the three categories of ICIs, we found that anti‐PD‐L1s might have a higher signal of hepatic failure than anti‐PD‐1s and anti‐CTLA‐4s; however, previous studies have indicated that anti‐CTLA‐4s and anti‐PD‐1s were more associated with a higher risk of high‐grade hepatotoxicity.^15^, ^16^ It seems that the strong signal of anti‐PD‐L1s in our study was mainly due to the strong signal of atezolizumab. For monotherapy, atezolizumab might show the strongest risk signal of hepatic failure. There has been one case of acute hepatic failure reported with atezolizumab^5^; however, it has been reported that ipilimumab, pembrolizumab, and nivolumab were more associated with liver damage.^17^, ^18^ Considering that atezolizumab was approved later and its adverse effects have only been identified in recent years, our findings may not be enough to conclude that atezolizumab has the highest risk of hepatic failure among all ICIs. Nevertheless, future clinical studies could pay more attention to hepatic failure following the administration of atezolizumab. Moreover, we compared ICI combination therapy with all other drugs, and the combination of nivolumab and ipilimumab was the most commonly reported and might have the strongest risk signal of hepatic failure. This combination therapy also showed a possibly higher association with hepatic failure than monotherapy, consistent with previous studies.^19^ Nivolumab plus ipilimumab, the first‐line treatment for advanced melanoma, can prolong patient survival.^20^ In order to balance the benefits and risks, clinicians may continue to choose this regimen with enhanced liver function monitoring and prophylactic treatment.

We compared the combination of ICIs and the most frequently used concomitant agents with ICI monotherapy. The combination of ICIs with other anti‐tumor agents, such as bevacizumab and paclitaxel, the first‐line regimen for advanced hepatocellular carcinoma and non‐small cell lung cancer, can prolong patient survival,^21^, ^22^ and the high‐risk signals of hepatic failure might be due to accumulation of the adverse effects of anti‐tumor agents. Furthermore, some analgesics might be used to relieve advanced cancer pain; however, acetaminophen, a hepatotoxic agent,^23^ was not suitable for concomitant use with ICIs as this combination caused cumulative hepatotoxicity. This finding warns clinicians to be cautious about combining other hepatotoxic drugs when patients are taking ICIs, and medications should be monitored and adjusted in time. In addition, several other agents with risk signals, such as prednisone, omeprazole, furosemide, and levothyroxine, might be used to treat ICI‐associated adverse events that have occurred, and we are far from clear that these agents can increase the risk of ICI‐associated hepatic failure, and it is debatable whether these signals have significance.

It has been reported that younger patients seem to be more associated with ICI‐associated serious adverse events,^24^ similar to our findings (age < 65 vs. age ≥ 65: ROR 1.35, 95% CI 1.13–1.60) (Table S4 and S5). Lower immunity in older patients might result in a lower effect of ICIs, as well as weaken immune‐related adverse events. The age difference might prompt increased vigilance for this adverse event in younger patients; however, whether age needs to be used as a criterion before treatment with ICIs is unclear and should be further investigated. For the gender subgroup, we found no risk signal, and it may not be necessary to consider gender in clinical practice.^25^, ^26^ A cohort study that aimed to investigate risk factors for post‐hepatectomy liver failure showed that 4.09% (171/4178) of patients with liver metastases developed liver failure after hepatectomy.^27^ However, in our study, only 0.76% (5/654) of patients with hepatic failure developed liver metastases.

We found that the median TTO of ICIs was 38 days and 72.30% of cases occurred within the first 3 months, as most cases were acute and subacute hepatic failure. According to the clinical diagnostic criteria for hepatic failure, acute hepatic failure has an acute onset and usually appears within 2 weeks, while subacute hepatic failure usually appears between 2 and 26 weeks. The main clinical symptoms are (1) extreme malaise or visible gastrointestinal symptoms, (2) rapid deepening of jaundice, (3) with or without hepatic encephalopathy, (4) bleeding, with other causes excluded.^28^ Clinicians may need to be vigilant for hepatic failure in the early stages of ICIs administration, especially in the first 3 months. Further research on risk prediction should also be conducted in the future to guide clinical identification and management of ICI‐associated hepatic failure.

Hepatic failure is rare but fatal. In a meta‐analysis that included 19,127 patients treated with ICIs, 8 patients developed fatal liver damage.^8^ In our study, the fatality proportion of ICI‐associated hepatic failure in the FAERS database was up to 68.65%; however, we were unable to obtain the fatality rate as the precise total number of patients with ICIs is not available. Some studies tended to report only fatal adverse events, and cases with milder symptoms were likely to be underreported, which might contribute to the high fatality proportion. Nonetheless, this high fatality proportion warns clinicians to strengthen liver function monitoring for early prophylactic and early treatment.

Our study has some limitations. Firstly, the FAERS database is a spontaneous reporting system, without strict regulation; therefore, data in the FAERS database are not as reliable as clinical trials and cohort studies. Secondly, this database does not cover all real‐world data and most mild cases may be underreported, which may have resulted in bias in our analysis. Thirdly, this database can neither identify causality for ICIs nor exclude cofactors, such as alcohol, hepatitis or liver disease, cardiopulmonary complications and decompensation, sepsis, and additional comorbidities. No subgroup analysis of the patients' primary diseases was performed. Fourthly, uncertainties in diagnostic classifications, as well as eventual outcomes, and others, pose significant restrictions. Fifthly, the total number of patients treated with ICIs was not available in the FAERS database; thus, it cannot be used to calculate incidence rates.

## 5 CONCLUSION

This study aimed to evaluate the association between ICIs and hepatic failure based on the FAERS database. ICIs had possible strong signals associated with hepatic failure. Most cases with hepatic failure occurred within the first 3 months after ICIs initiation and had a poor outcome, which suggests that liver function monitoring needs to be enhanced after the administration of ICIs.

## AUTHOR CONTRIBUTIONS

**Ye Xu:** Data curation (lead); methodology (equal); visualization (lead); writing – original draft (lead); writing – review and editing (equal). **cilin Yan:** Data curation (supporting); software (lead). **Ying Zhao:** Supervision (equal); writing – review and editing (equal). **Dandan Li:** Supervision (equal); writing – review and editing (equal). **mingxing Guo:** Supervision (equal); writing – review and editing (equal). **xiangli Cui:** Conceptualization (equal); resources (equal); supervision (equal); writing – review and editing (equal).

## FUNDING INFORMATION

This work was supported by Beijing Hospital Authority (PG2020002) and Beijing Science and Technology Planning Project (KJ2022CX039).

## CONFLICT OF INTEREST STATEMENT

All authors declare that they have no conflicts of interest.

## Supporting information


Table S1.
Table S2.
Table S3.
Table S4.
Table S5.
Figure S1.
Figure S2.

## Data Availability

All data are available within the manuscript and supplemental materials, and FAERS database was available at website (shown in the data source): https://open.fda.gov/data/faers/.
