# Supplementary material for: Hepatic failure associated with immune checkpoint inhibitors: An analysis of the Food and Drug Administration Adverse Event Reporting System database
Source: Cancer Med. 2023 Feb 3;12(8):9167–74. doi: 10.1002/cam4.5655 (PMC10166896; doi:10.1002/cam4.5655)
Supplement: Supplementary file 1 — Table S1. Table S2. Table S3. Table S4. Table S5. Figure S1. Figure S2. [file CAM4-12-9167-s001.docx]

**Hepatic failure associated with immune checkpoint inhibitors: An analysis of the Food and Drug Administration Adverse Event Reporting System (FAERS) database**

**Supplementary Information**

**1 Abbreviations**

**2 Supplemental tables**

Table S1 Drug information for 8 FDA approved ICIs

Table S2 Summary of major algorithms used for signal detection

Table S3 Number of death cases and fatality proportion of ICI-associated hepatic failure

Table S4 Signals of ICI-associated hepatic failure in the different subgroups of genders and ages

Table S5 Subgroup comparison of ICI-associated hepatic failure (Female vs. Male; Age＜65 vs. Age≥65; Anti-PD-L1s vs. Anti-PD-1s vs. Anti-CTLA-4s)

**3 Supplemental figures**

Fig. S1 Process of selecting cases of ICI-associated hepatic failure from the FAERS database

Fig. S2 Signals of concomitant used ICIs with other agents associated hepatic failure

**Abbreviations:**

AIH: autoimmune hepatitis

CTLA-4: cytotoxic T lymphocyte associated protein 4

FAERS: Food and Drug Administration Adverse Event Reporting System

ICIs: immune checkpoint inhibitors

IC: information component

IC_025_: the lower limit of the 95% two-sided confidence interval of the IC

MedDRA: Medical Dictionary for Regulatory Activities

PD-1: programmed cell death protein 1

PD-L1: programmed cell death 1 ligand 1

PTs: preferred terms

ROR: reporting odds ratio

ROR_025_: the lower limit of the 95% two-sided confidence interval of the ROR

SMQ: Standardised MedDRA Queries

TTO: time to onset

Table S1 Drug information for 8 FDA approved ICIs

| Generic names | Brand names | Approval year | Target |
| --- | --- | --- | --- |
| Nivolumab | Opdivo,BMS-986298,Opdyta,BMS-936558 | 2014 | PD-1 |
| Pembrolizumab | Keytruda,MK-3475 | 2014 | PD-1 |
| Cemiplimab | Libtayo,SAR439684,REGN2810 | 2018 | PD-1 |
| Atezolizumab | Tecentriq,MPDL3280A | 2016 | PD-L1 |
| Avelumab | Bavencio,MSB0010718C | 2017 | PD-L1 |
| Durvalumab | Imfinzi,MEDI4736 | 2017 | PD-L1 |
| Ipilimumab | Yervoy,BMS-734016 | 2011 | CTLA-4 |
| Tremelimumab | Ticilmumab | - | CTLA-4 |

Table S2 Summary of major algorithms used for signal detection

| Algorithms | Equation | Criteria | Code |
| --- | --- | --- | --- |
| ROR | ROR=(a/b)/(c/d) | ROR025>1,N≥3 | ROR=(a/b)/(c/d) |
|  | 95%CI=eln(ROR)±1.96(1/a+1/b+1/c+1/d)^0.5 |  | ROR025 = np.exp(np.log(add['ROR']) - 1.96 * (1. / a + 1. / b + 1. / c + 1. / d) ** 0.5) |
|  |  |  | ROR975 = np.exp(np.log(add['ROR']) + 1.96 * (1 / a + 1 / b + 1 / c + 1 / d) ** 0.5) |
| BCPNN | IC=log2a(a+b+c+d)/((a+c)(a+b)) | IC025>0 | IC = np.log2(a * (a + b + c + d) / ((a + c) * (a + b))) |
|  | 95%CI=eln(IC)±1.96(1/a+1/b+1/c+1/d)^0.5 |  | IC025 = np.exp(np.log(add['IC']) - 1.96 * (1 / a + 1 / b + 1 / c + 1 / d) ** 0.5) |
|  |  |  | IC975 = np.exp(np.log(add['IC']) + 1.96 * (1 / a + 1 / b + 1 / c + 1 / d) ** 0.5) |

BCPNN, Bayesian confidence propagation neural network; CI, confidence interval; IC, information component; IC_025_, the lower limit of the 95% two-sided CI of the IC; N, the number of co-occurrences; ROR, reporting odds ratio; ROR_025_, the lower limit of the 95% two-sided CI of the ROR; *a: number of reports containing both the suspect drug and the suspect adverse drug reaction; b: number of reports containing both the suspect drug and other adverse drug reactions (except the event of interest); c: number of reports containing both other medications (except the drug of interest) and the suspect adverse drug reaction; d: number of reports containing other medications and other adverse drug reactions.

Table S3 Number of death cases and fatality proportion of ICI-associated hepatic failure

| Drug | Fatal cases, n (%) | Non-fatal cases, n ( %) |
| --- | --- | --- |
| **Monotherapy** |  |  |
| **Anti-PD-1** | 325 (68.42%) | 150 (31.58%) |
| Nivolumab* | 235 (69.32%) | 104 (30.68%) |
| Pembrolizumab* | 91 (65.94%) | 47 (34.06%) |
| Cemiplimab | 1 (100%) | 0 |
| **Anti-PD-L1** | 109 (68.99%) | 48 (30.57%) |
| Atezolizumab* | 88 (66.17%) | 45 (33.83%) |
| Avelumab | 8 (88.89%) | 1 (11.11%) |
| Durvalumab | 14 (77.78%) | 3 (17.65%) |
| **Anti-CTLA-4** | 95 (61.69%) | 59 (38.31%) |
| Ipilimumab* | 93 (61.59%) | 58 (38.41%) |
| Tremelimumab | 2 (66.67%) | 1 (33.33%) |
| **Combination therapy** |  |  |
| Nivolumab+Ipilimumab* | 76 (59.84%) | 51 (40.16%) |
| Durvalumab+Tremelimumab | 2 (66.67%) | 1 (33.33%) |
| **All Drug** | 449 (68.65%) | 204 (31.24%) |

Note: ***Pearson's chi-squared test was performed for ICIs with case numbers greater than 20, and the result showed no statistical difference (*P*=0.282).

Table S4 Signals of ICI-associated hepatic failure in the different subgroups of genders and ages

| Groups  (ICIs vs. all other drugs) | a (N) | b | c | d | ROR  (ROR_025_-ROR_975_) | IC  (IC_025_-IC_975_) |
| --- | --- | --- | --- | --- | --- | --- |
| **All ICIs** | 654 | 119736 | 17800 | 9509397 | 2.92 (2.70-3.16) | 1.51 (1.39-1.63) |
| **Gender** |  |  |  |  |  |  |
| Female | 218 | 40214 | 8024 | 5061242 | 3.42 (2.99-3.91) | 1.74 (1.52-1.99) |
| Male | 382 | 65361 | 6906 | 3277610 | 2.77 (2.50-3.08) | 1.42 (1.28-1.57) |
| **Age** |  |  |  |  |  |  |
| ＜65 | 274 | 37397 | 8980 | 3323999 | 2.71 (2.40-3.06) | 1.41 (1.25-1.59) |
| ≥65 | 251 | 46106 | 3795 | 1973244 | 2.83 (2.49-3.22) | 1.44 (1.26-1.63) |

Note: a: number of reports containing both the ICIs and hepatic failure; b: number of reports containing both the ICIs and all other adverse events (except hepatic failure); c: number of reports containing both all other drugs (except ICIs) and hepatic failure; d: number of reports containing all other drugs and all other adverse events.

CI: confidence interval; IC: information component; IC_025_: the lower limit of the 95% two-sided confidence interval of the IC; IC_075_: the upper limit of the 95% two-sided confidence interval of the IC; ROR: reporting odds ratio; ROR_025_: the lower limit of the 95% two-sided confidence interval of the ROR; ROR_075_: the upper limit of the 95% two-sided confidence interval of the ROR.

Table S5 Subgroup comparison of ICI-associated hepatic failure (Female vs. Male; Age＜65 vs. Age≥65; Anti-PD-L1s vs. Anti-PD-1s vs. Anti-CTLA-4s)

| Subgroup comparison | a (N) | b | c | d | ROR  (ROR_025_-ROR_975_) |
| --- | --- | --- | --- | --- | --- |
| **Gender** |  |  |  |  |  |
| Female vs. male | 218 | 40214 | 382 | 65361 | 0.93 (0.79-1.10) |
| **Age** |  |  |  |  |  |
| ＜65 vs. ≥65 | 274 | 37397 | 251 | 46106 | 1.35 (1.13-1.60) |
| **ICIs categories** |  |  |  |  |  |
| Anti-PD-L1s vs. Anti-PD-1s | 158 | 22933 | 475 | 90710 | 1.32 (1.10-1.58) |
| Anti-CTLA-4s vs. Anti-PD-1s | 154 | 24399 | 475 | 90710 | 1.21 (1.004-1.45) |
| Anti-PD-L1s vs. Anti-CTLA-4s | 158 | 22933 | 154 | 24399 | 1.09 (0.87-1.36) |

Note: a: number of reports containing both ICIs and hepatic failure in one subgroup; b: number of reports containing both ICIs and all other adverse events (except hepatic failure) in one subgroup; c: number of reports containing both ICIs and hepatic failure in another subgroup; d: number of reports containing both ICIs and all other adverse events in another subgroup.

CI: confidence interval; ROR: Reporting Odds Ratio; ROR_025_: the lower limit of the 95% two-sided confidence interval of the ROR; ROR_075_: the upper limit of the 95% two-sided confidence interval of the ROR.


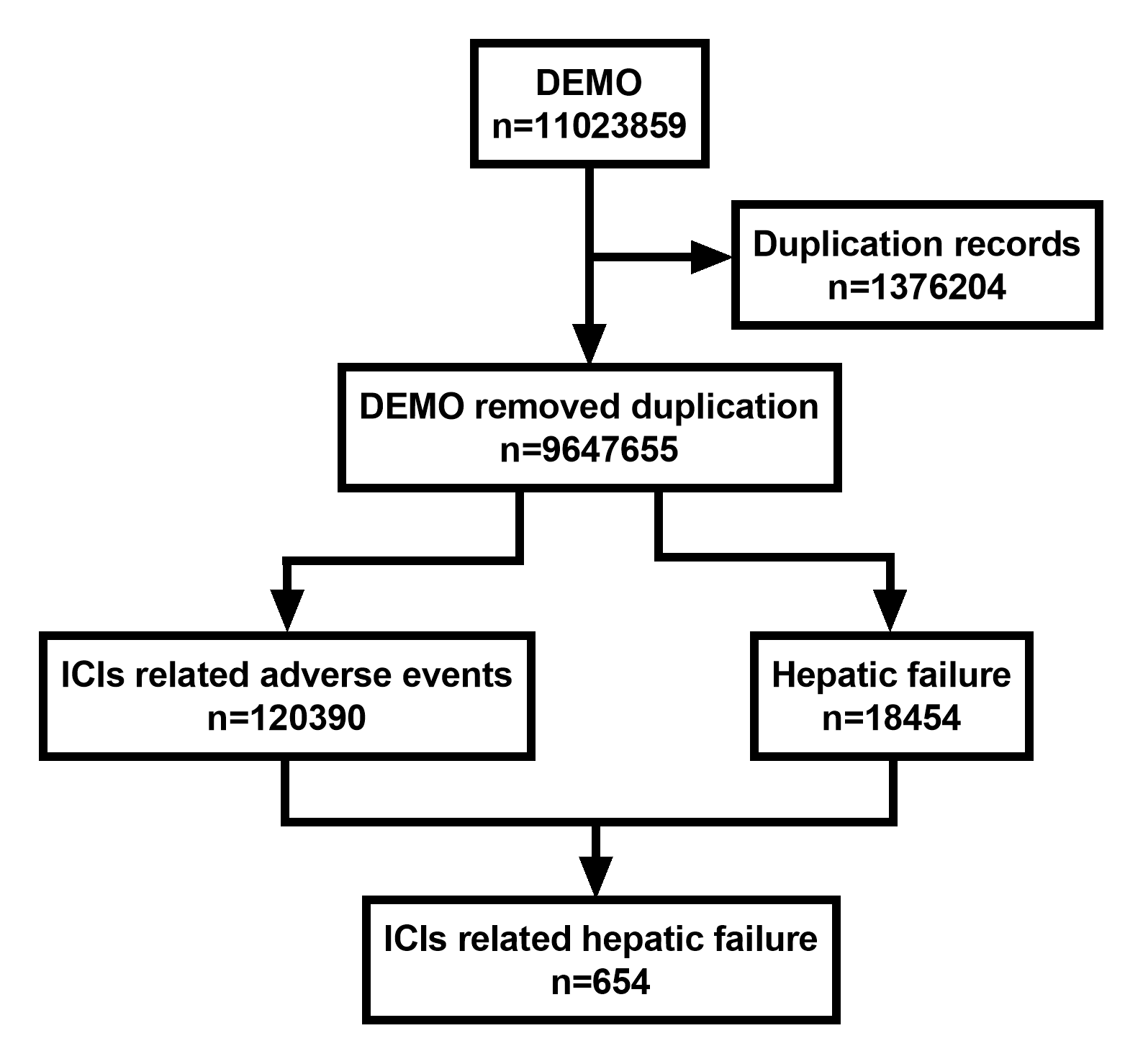


Fig. S1 Process of selecting cases of ICI-associated hepatic failure from the FAERS database

DEMO: demographic and administrative information; FAERS: Food and Drug Administration Adverse Event Reporting System; ICIs: immune checkpoint inhibitors.


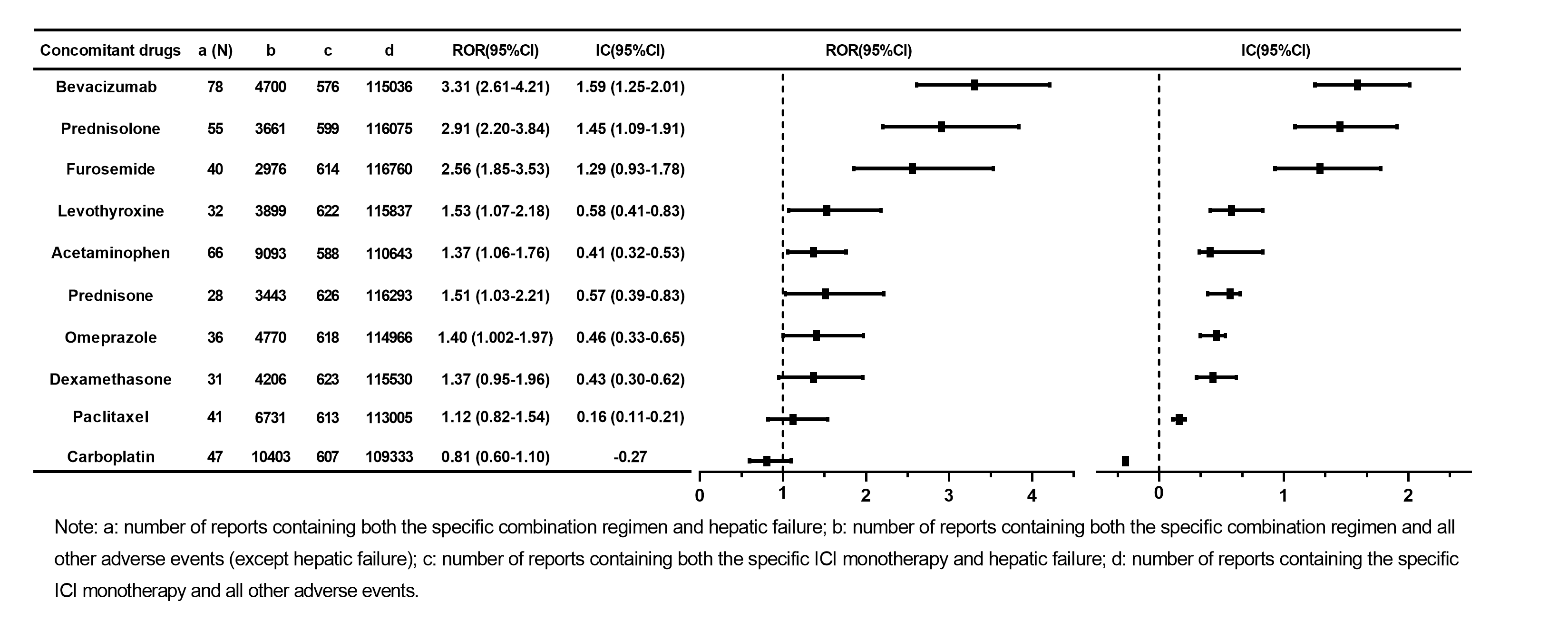


Fig. S2 Signals of concomitant used ICIs with other agents associated hepatic failure

95% CI: the 95% two-sided confidence interval; IC: information component; ROR: reporting odds ratio.
